# Mutualists or parasites? Context-dependent influence of symbiotic fly larvae on carnivorous investment in the Albany pitcher plant

**DOI:** 10.1098/rsos.160690

**Published:** 2016-11-23

**Authors:** Samuel J. Lymbery, Raphael K. Didham, Stephen D. Hopper, Leigh W. Simmons

**Affiliations:** 1 Centre for Evolutionary Biology, School of Animal Biology, The University of Western Australia, 35 Stirling Highway, Crawley, Western Australia 6009, Australia; 2 School of Animal Biology, The University of Western Australia, 35 Stirling Highway, Crawley, Western Australia 6009, Australia; 3 Centre for Environment and Life Sciences, CSIRO Land and Water, Underwood Avenue, Floreat, Western Australia 6014, Australia; 4 Centre of Excellence in Natural Resource Management, and School of Plant Biology, The University of Western Australia, Albany, Western Australia 6330, Australia

**Keywords:** carnivorous plants, infauna, insect–plant interaction, nutrient limitation, resource allocation, phenotypic plasticity

## Abstract

Carnivorous plants allocate more resources to carnivorous structures under nutrient-limited conditions, and relative investment can also be influenced by animals (infauna) that live in association with these plants and feed on their prey. We investigated these effects within a population of the pitcher plant *Cephalotus follicularis* containing varying densities of larvae of the fly *Badisis ambulans*. For plants with a relatively high proportion of adult pitchers, increasing larval density was associated with lower relative leaf allocation to new pitcher buds. For plants with relatively few adult pitchers, however, there was greater relative leaf allocation to pitcher buds with increasing larval density. In a field experiment, there was no significant effect of experimental larval presence or absence on the change in carbon-to-nitrogen (C/N) ratio of plants. Although the direction of the correlation between *B. ambulans* larvae and relative investment in carnivorous and non-carnivorous structures depends on the relative number of mature structures, whether the larvae enhance or reduce nutrient stress under different conditions remains unclear. The change in C/N was, however, less variable for pitchers that contained larvae, suggesting a stabilizing effect. Eighteen of 52 experimental pitchers were damaged by an unknown species, causing the pitcher fluid to drain. These pitchers were significantly more likely to survive if they contained larvae. These results suggest that the relationship between infauna and host varies with the initial resource status and environmental context of the host plant.

## 1. Introduction

Carnivorous plants typically respond to changing nutrient conditions by changing relative investment in different structures. The cost–benefit model of plant carnivory [1] assumes that carnivory should be favoured only when the productivity benefits from added nutrients outweigh the energetic costs of producing carnivorous structures, which are inefficient for other functions such as light-harvesting. This will be the case only when nutrients, rather than other factors such as light and/or water, limit productivity. Furthermore, plants under more nutrient-limited conditions should allocate a greater proportion of their resources to carnivorous structures [2–5]. These responses, however, may not be determined only by environmental nutrient availability, but also by relationships with symbiotic animal species.

Pitcher plants frequently play host to a variety of animal species (infauna). The pitchers of these plants provide shelter and nutrients for any animals that are able to resist the digestive properties of the pitcher fluid. As a result, the majority of pitcher plant species investigated to date have contained infauna [6–16]. By feeding on prey items captured by the pitchers, infauna can affect prey assimilation rates, either positively [10,17–20] or negatively [19] and therefore plant nutrient status and resource allocation strategies. We would therefore expect an association between the presence and abundance of infauna and (i) the relative proportion of nutrients in plant tissues and (ii) the relative number of carnivorous and non-carnivorous structures. While we might expect mutualistic infauna to stimulate a reduction in carnivorous investment by reducing nutrient stress, alternative responses are also possible [17]. Any examination of the nature (mutualism or parasitism) of the plant–infauna relationship should therefore not be limited to an examination of the effect of infauna on carnivorous investment, but should also be coupled with an examination of the nutrient status of the host.

**Figure 1.**
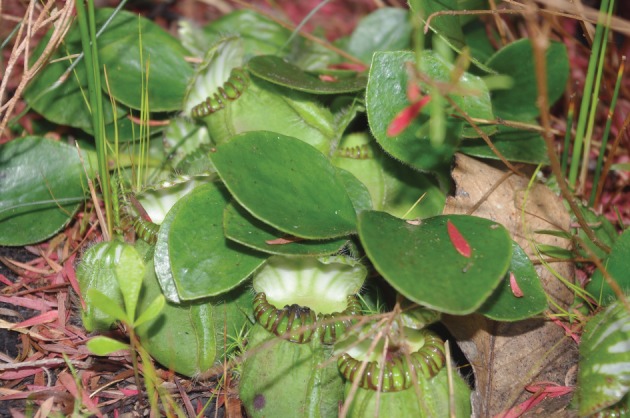
The Albany pitcher plant *Cephalotus follicularis,* showing carnivorous and non-carnivorous leaves. Taken in June 2014 in Two Peoples Bay Nature Reserve, Western Australia. Photo credit: Jennifer Lymbery.

We examined the relationship between infauna density and (i) the relative number of new carnivorous structures and (ii) nutrient status of plant tissues in the Albany pitcher plant, *Cephalotus follicularis* (figure 1), a vulnerable species (as listed by the International Union for the Conservation of Nature) which is phylogenetically distinct from other pitcher plants and found only in the margins of peat swamps in the Southwest Australian Floristic region [21–26]. *Cephalotus follicularis* contains fewer infauna species than many other pitcher plants, and by far the most common macroscopic inhabitants of the pitchers are the larvae of the micropezid fly *Badisis ambulans* [22,27]. The larvae of these flies live in the pitchers of *C. follicularis*, and scavenge the prey items captured by the plant. The adults are flightless ant mimics, but much of the ecology of both adults and larvae remains unknown [27–30]. In addition, almost nothing is known of possible interactions between *B. ambulans* and other associated invertebrates, or the effects that these interactions (if they occur) may have on *C. follicularis*. We examined whether the density of *B. ambulans* within pitchers was associated with relative investment in carnivorous structures by the plant (as measured by relative numbers of new pitcher buds), and/or changes in plant nutrient status (carbon-to-nitrogen (C/N) ratio) that might be influenced by altered assimilation of nutrients from prey.

## 2. Material and methods

### 2.1. Site description

Fieldwork was performed in a population of *C. follicularis* at Two Peoples Bay Nature Reserve, Western Australia. The population occurs along a shallow slope above a seepage zone, with areas of waterlogged soil present throughout the year. All fieldwork was completed in accordance with Department of Parks and Wildlife Western Australia (DPaW) permits SW016040 and SF009718 (licences to take flora and fauna for scientific purposes, respectively).

### 2.2. Carnivorous investment

Plants were selected along the full length of the field site by proceeding along a line parallel to Two Peoples Bay Road and selecting visible plants at 2 m intervals until 113 plants had been sampled, with the restriction that plants were identifiable as individuals rather than as clonal patches. For each plant, we recorded the number of pitcher buds and the number of non-carnivorous leaf buds. Buds were recorded instead of mature pitchers and leaves for two reasons. First, *B. ambulans* larvae could not have influenced the expression of the mature pitchers in which the larvae lived, because the pitchers were by necessity already present when the larvae arrived. Second, new buds, which were less likely to be present when the eggs were laid (courtship and egg-laying are only thought to occur in summer, at least four months before this study was conducted; D. Yeates 2014, personal communication), were also less likely to influence the oviposition behaviour of *B. ambulans*.

The number of larvae in each plant was determined by draining all mature pitchers, using modified glass Pasteur pipettes that had been cut above the terminal sections to allow the passage of larger pieces of debris [21]. The effectiveness of this method was assessed by examining the interior of the pitchers with an auriscope. Larval density was calculated for each plant as number of flies per unit volume rather than as number of flies per pitcher, because the size of the pitcher theoretically determines the quantity of prey it can hold and digest. Pitcher volume was calculated from measurements of height and width, assuming a cylindrical shape. For each plant, the possible influential variables of shading and proportion of adult pitchers (number of adult pitchers divided by total number of carnivorous and non-carnivorous adult leaves) were also recorded. We used the proportion of adult pitchers rather than absolute number of adult pitchers, because the former weights pitcher frequency by total plant size (as approximated by the total number of leaves), and therefore provides a better representation of the plant's ability to supply nutrients from prey given the quantity of tissue that requires provisioning. Shading may also be important, because light availability might influence the relative pay-off of investing in carnivorous versus photosynthetic leaves, and might also influence visitation rates from insects and other invertebrates. Shading was estimated visually as the proportion of each plant that was covered by surrounding vegetation when looking down from above. For consistency of estimates, the same person (S.J.L.) estimated shading for all plants.

The relationship between larval density and proportion of pitcher buds, including the possible effects of shading and proportion of adult pitchers, was examined using a generalized linear model (GLM) with binomially distributed errors. Predictor variables were standardized (i.e. mean-centred and divided by 2 standard deviations) prior to analyses [31], and we tested for collinearity by calculating variance inflation factors (VIFs). All VIFs were less than three (proportion of adult pitchers = 1.04, shading = 1.04 and larval density = 1.01), so collinearity was weak and would not bias model interpretation [32]. Four possible link functions (logit, probit, cauchit and complementary log–log) were assessed by comparing Akaike information criterion (AIC) scores and Akaike weight [33]. The cauchit link function produced the highest weight (Akaike weight = 0.55, with the logit link also within two AIC units), and this model was used in subsequent analyses. Models with all possible combinations of parameters were compared in the same way as described for the comparison of link functions, and we selected the top model as long as the null model was not within two AIC units [31,34,35]. If the null model was within two AIC units of the top model, then this was interpreted as a lack of strong evidence for significant effects of the predictor variables. Overdispersion was assessed by comparing the residual deviance to the residual degrees of freedom, and was not considered to be significant if this ratio was less than two and the dispersion parameter was close to one [34]. All statistical analyses were performed in R [36].

It is plausible that the proportion of adult pitchers might increase simply as a function of plant age, but we were not able to directly measure age in *C. follicularis*. We could use total plant size (i.e. total number of adult carnivorous and non-carnivorous leaves, combined) as a proxy for plant age, but we were not able to include this in the same GLM analyses with proportion of adult pitchers owing to their high inherent collinearity. Therefore, we conducted an alternative sensitivity analysis in which we included ‘plant age’ (i.e. total plant size) instead of proportion of pitchers in the GLM and compared model fit using AIC comparisons. Although ‘plant age’ was included in the top model, it was not a significant predictor of carnivorous investment in new pitcher buds (electronic supplementary material, tables S1 and S2). We therefore retained the original analysis with proportion of adult pitchers rather than with age, as proportion of adult pitchers had greater explanatory power than the variable ‘plant age’.

### 2.3. Nutrient status experiment

At the field site, 50 experimental plants were selected and had their pitchers drained as described above. *B. ambulans* larvae were removed, and the fluid was replaced. Plants remained empty for four weeks, after which 25 were randomly allocated to the ‘fly’ treatment using a random number generator, and each plant had 10 *B. ambulans* larvae placed in one pitcher. The remaining 25 plants remained empty of larvae (‘no-fly’ treatment). Adult *B. ambulans* are not known to be seasonally active during the time period the study was conducted (April to June; D. Yeates 2014, personal communication), and it is therefore unlikely that adult flies subsequently laid eggs in experimental plants. Pre-experiment plant tissue samples were taken by removing half the lid of each experimental pitcher. After four more weeks, examination of the plants revealed that 18 of the experimental pitchers had small holes bored in the base, so that the fluid had drained. The damage had presumably been caused by some unknown animal species. Of these damaged pitchers, eight had subsequently withered, and the remainder had been drained but were still alive. A Fisher's exact test was used to test for differences between fly and no-fly treatments in terms of the proportion of damaged pitchers that had withered. All of the damaged pitchers (withered and non-withered) were excluded from the nutrient analyses, together with one pitcher which was destroyed by other means. After a further four weeks, post-experiment plant tissue samples were collected.

The nutrient status of the experimental plants was assessed, using the C/N ratio. In non-carnivorous plants, C/N is related to environmental conditions such as soil nutrient content. Because plants use light to fix C, and subsequently combine this C with nutrients taken from the soil, a lower C/N generally indicates an increase in relative nutrient availability [32,37]. While carnivorous plants capture and digest prey as a means of supplementing nutrient uptake, carnivory is not considered a form of true heterotrophy because they do not generally take up C from prey (but see [1,7,38–43]). Consequently, more efficient plants (in terms of carnivory) should exhibit lower C/N ratios [32,37,41]. If infauna affect carnivorous efficiency, therefore, then they should also alter C/N.

Pre- and post-experiment samples were oven-dried to a constant weight at 60°C, and ground to a fine powder, using a ball-mill grinder. Between 1.00 and 1.20 mg of each sample was weighed and packed into small tin capsules. These were analysed for C/N using an automated nitrogen carbon analyser (Sercon™ 20–22 isotope ratio mass spectrometer). The change in C/N for each plant was compared between ‘fly’ and ‘no-fly’ treatments using a GLM (Gaussian error distribution), with shading, proportion of adult pitchers and pre-experiment fly density included as covariates. Prior to analyses, one more sample was removed as an outlier, giving a final sample size of *n* = 15 per larval treatment (30 plants in total). Predictors were standardized prior to analyses. Once again, we calculated VIFs for predictor variables, and because all were less than three (proportion of adult pitchers = 1.16, shading = 1.05, pre-experiment larval density = 1.12, larval treatment = 1.11) collinearity was not considered to influence model interpretation [32]. Model comparison was performed as described for the carnivorous investment analysis. Homoscedasticity was assessed by examining the plot of residuals against fitted values, and normality was assessed by examining the distribution of the model residuals. As for the carnivorous investment analyses, we conducted a sensitivity analysis in which we included ‘plant age’ instead of proportion of pitchers. Models including the variable ‘plant age’ were, however, within two AIC units of the null model (electronic supplementary material, table S3). The original analysis, including proportion of adult pitchers rather than ‘plant age’, was therefore retained, as for the carnivorous investment analysis. Identical analyses were also performed for plant total N content and *δ*^15^N isotope ratio, because both of these metrics might also be expected to change as a result of infauna activity. Total N is essentially an alternative measure to C/N, and would be expected to yield similar information. *δ*^15^N typically increases up the food chain, and plants gaining a greater proportion of total N from insect prey rather than the soil should have a higher *δ*^15^N [7,44–47]. Note, however, that *δ*^15^N was measured only in post-experiment samples, because the mass of tissue required was too great for pre-experiment measurements. It was therefore not possible to analyse change in *δ*^15^N across the course of the experiment.

## 3. Results

### 3.1. Carnivorous investment

**Figure 2.**
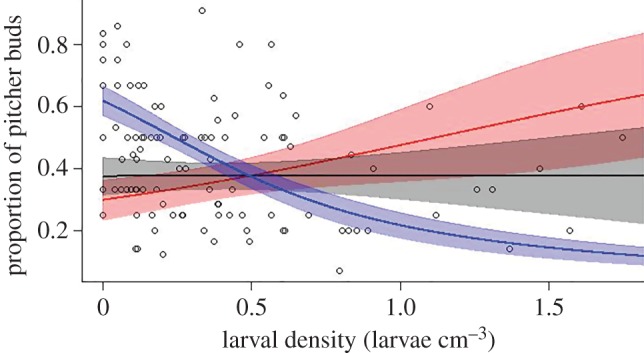
The effect of the interaction between proportion of adult pitchers and *Badisis ambulans* density on carnivorous investment in new pitcher buds in *Cephalotus follicularis*. Fitted lines (±1 s.e.) represent the predictions from a binomial GLM employing the Cauchit link function, which accounts for the significant interaction effect of proportion of adult pitchers, at 0.2 (red), 0.4 (black) and 0.9 (blue) proportions of adult pitchers (arbitrary levels selected for illustrative purposes). Although the top model also included an effect of shading, this effect did not influence the interaction between the proportion of adult pitchers and larval density, and has been removed for illustrative purposes.

The top model included larval density, the proportion of adult pitchers, the interaction of these two variables and shading as predictors, and the null model was not within two AIC units of the top model (table 1). We confirmed that there was no overdispersion of model residuals (ratio of residual deviance to residual degrees of freedom = 1.95, dispersion parameter = 1.48). Larval density was significantly associated with the proportion of pitcher buds, but this association was dependent on the proportion of adult pitchers on the plant (table 2 and figure 2). When the proportion of adult pitchers was low (less than 0.4) increasing larval density was associated with an increasing proportion of pitcher buds (figure 2). When the proportion of adult pitchers was high (greater than 0.4) increasing larval density was associated with a decreasing proportion of pitcher buds (figure 2). The ‘shading’ term did not affect the relationship between larval density (the main predictor of interest in this study) and carnivorous investment. Higher levels of shading, however, were positively associated with the proportion of pitcher buds (table 2).

**Table 1.** Model comparison of the drivers of variation in the proportion of pitcher buds on *Cephalotus follicularis* plants. Akaike's information criterion (AIC), number of model parameters (*k*) and model weights for binomial generalized linear models within two AIC units of the top model (plus the full model fit for comparison). Predictors were the proportion of adult pitchers (PP), proportion shading by vegetation (shading) and density of *Badisis ambulans* larvae (density). Predictors were standardized prior to model comparisons.

predictors	*k*	AIC	ΔAIC	Akaike weight
PP + density + shading + PP : density	5	286.35	0.00	0.42
PP + density + shading + PP : density + density : shading	6	287.63	1.28	0.22
PP + density + PP : density	4	288.09	1.74	0.18
PP + density + shading + PP : density + PP : shading	6	288.13	1.78	0.17
full model: PP × shading × density	8	288.53	2.18

**Table 2.** Results of the best-fit binomial GLM testing the effects of the proportion of adult pitchers (PP), density of the larvae of *Badisis ambulans* (density) and proportion of shading by neighbouring vegetation (shading) on the proportion of pitcher buds produced by *Cephalotus follicularis*. See table 1 for model comparisons.

predictor	coefficient estimate	s.e.	*z*	*p*
density	−1.02	0.31	−3.34	0.001*
PP	0.32	0.21	1.58	0.112
shading	0.40	0.20	1.98	0.048*
density × PP	−1.36	0.46	−2.97	0.003*

Asterisks denote significant *p*-values.

### 3.2. Nutrient status experiment

The mean change in the C/N ratio between pre- and post-experiment samples was negative for both ‘fly’ and ‘no-fly’ plants, although more so for ‘fly’ plants (fly: −14.67 ± 3.82; no-fly: −6.31 ± 8.10; table 3). The top models included the effects of proportion of adult pitchers and density of larvae on the change in C/N ratio, but these models were within two AIC units of the null model (table 4). This was interpreted as a lack of strong evidence for significant predictor effects on the response variable. Model residuals did not show heteroscedasticity or non-normality, but it was evident that the change in C/N was more variable for plants without larvae (table 3). Identical model results were obtained for total N content (electronic supplementary material, tables S4 and S5; table S6 for mean total N values) and *δ*^15^N (electronic supplementary material, tables S7 and S8; table S9 for mean *δ*^15^N values) as response variables, with top models within two AIC units of null models.

**Table 3.** Mean (± s.e.) carbon to nitrogen ratios of *Cephalotus follicularis* pitchers before and eight months after experimental manipulation of *Badisis ambulans* presence/absence. ‘Fly’ pitchers had all *B. ambulans* larvae removed and then 10 added to a single pitcher, whereas ‘no fly’ plants had all larvae removed and none added.

	before	after
fly	104.40 ± 3.47	89.73 ± 3.82
no fly	103.17 ± 3.92	96.86 ± 8.10

**Table 4.** Model comparison of the drivers of change in the carbon-to-nitrogen ratio of *Cephalotus follicularis* tissue from before to eight weeks after experimental manipulation of *Badisis ambulans* larval presence/absence. Akaike's information criterion (AIC), number of model parameters (*k*) and model weight scores for linear models within two AIC units of the top model (plus the full model fit for comparison). Predictors were the proportion of adult pitchers (PP), proportion shading by vegetation (shading), pre-experiment density of larvae (density) and the experimental presence/absence of larvae (treatment). Predictors were standardized prior to model comparisons.

predictors	*k*	AIC	ΔAIC	Akaike weight
PP	2	279.65	0.00	0.30
PP + density	3	280.12	0.46	0.24
null (intercept only)	1	280.41	0.75	0.21
PP + shading	3	281.21	1.56	0.14
shading	2	281.63	1.98	0.11
full model: PP × shading × density × treatment	16	291.86	12.21	

A total of 18 of the 50 experimental plants had holes bored in the sides by an unknown animal, nine in each of the ‘fly’ and ‘no-fly’ treatments. Of the damaged pitchers, a significantly greater proportion had withered in the ‘no-fly’ treatment (0.67) than in the ‘fly’ treatment (0.11; Fisher's exact test *p* = 0.049).

## 4. Discussion

Observational data showed that the association between the density of *B. ambulans* and investment in new carnivorous structures in *C. follicularis* depends on relative investment in existing mature pitcher structures. Our experimental manipulation, however, did not reveal any significant effect of larvae on the change in plant C/N, despite the direction of the difference between fly and no-fly plants being consistent with a mutualistic effect. Whether the association between larvae and the proportion of pitcher buds indicates nutritional mutualism or parasitism is therefore difficult to determine with complete confidence. The power of the nutrient status experiment was compromised owing to damage caused to experimental pitchers by an unknown animal species, and replication under more controlled conditions is potentially required in future studies. This unexpected damage, however, provided an additional and intriguing result, namely that larvae apparently enhance pitcher survival after damage. Together, our results suggest that the net effect of infauna such as *B. ambulans* on their hosts can change substantially across environmental and resource gradients.

### 4.1. Carnivorous investment and larval density

If *C. follicularis* responds to environmental conditions in a similar way to other carnivorous plant species, then the most likely explanation for lower investment in carnivorous structures is lower nutrient limitation [2–5]. If this is the case, then increasing larval density appears to be associated with decreased nutrient limitation in plants that had a greater proportion of adult pitchers, but increased nutrient limitation in plants that had few adult pitchers. If a causal relationship was to be confirmed with a manipulative experiment, this would represent a switch in relative mutualistic versus parasitic influences of *B. ambulans* on *C. follicularis* dependent on the resource status of the host plant.

One possible explanation for context dependence in infaunal influences on host plant investment in carnivorous structures is the relative balance between enhancement of nutrient assimilation by the host versus removal of total available nutrients by infaunal species. In many pitcher plant–infauna systems, it is assumed that breakdown of food by infauna enhances the ability of the plant to digest prey. However, because infauna also remove a certain amount of nutrients by feeding on prey items themselves, a nutritional mutualism will result only when digestive enhancement outweighs the reduction in total nutrients. In this system, this may be the case when plants have proportionally many pitchers and food capture is high (relative to the size of the plant), but not when plants have proportionally few pitchers and food capture is low.

While these results suggest that infauna can influence relative investment in carnivorous and non-carnivorous structures, our data were by their nature correlational. Experimental approaches are necessary to confirm causal effects. Furthermore, because pitcher buds may abort before they develop into adult carnivorous structures, it would be useful to perform a long-term study in which the net change in adult pitchers was measured from the beginning to the end of the manipulation. In this case, such a long-term experiment was not possible, unfortunately.

In addition, it would be valuable to confirm the inverse relationship between carnivorous investment and nutrient stress in *C. follicularis*. Although many studies have demonstrated this effect [2–5], positive correlations are not unheard of [17,48]. Furthermore, the way that carnivorous plants respond to levels of soil-derived nutrients may not be identical to responses to prey-derived nutrients. For example, while Bott *et al*. [2] and Meyer *et al*. [4] showed that increases in soil nutrient levels reduced carnivorous investment in the pitcher plant *Sarracenia purpurea*, Bazile *et al*. [17] showed that the infaunal ant *Camponotus schmitzii* reduced nutrient stress in *Nepenthes bicalcarata* but apparently promoted investment in carnivory by enhancing the levels of prey-derived nutrients. If this were the case for *C. follicularis*, then the association of *B. ambulans* with nutrient stress might still depend on the proportion of adult pitchers, but in the reverse direction to that considered above. It is for this reason that the manipulative nutrient status experiment was also included in this study, but the results of this experiment were, unfortunately, inconclusive (see below). Finally, although we used larval density as a function of total pitcher volume in these analyses to account for a plant's ability to capture prey, it might also be useful in future studies to include a direct measure of total prey biomass in the models, to further account for possible correlations between larval density and prey capture.

Finally, with regards to the carnivorous investment section of this study, it is interesting to note that shading had a marginally significant positive effect on the proportion of pitcher buds. Why this should be the case is not clear. In theory, reduced light availability might be expected to prompt increased investment in non-carnivorous photosynthetic leaves rather than in new pitchers. It is, however, possible that the variable ‘shading’ was correlated with some other, undetected variable, such as invertebrate activity or trap effectiveness. Because shading did not influence the effect of larvae on carnivorous investment, this effect is not of primary interest to this study, but might provide interesting opportunities for future work.

### 4.2. Nutrient status and larval presence

Our experimental manipulation of infaunal fly density did not reveal a significant effect of larvae on the change in nutrient status of *C. follicularis*. While the decrease in plant C/N ratio was greater in the ‘fly’ treatment than the ‘no fly’ treatment, this effect was not statistically significant. Unfortunately, our experiment suffered substantially from interference by an unknown animal species, and the reduction in power caused by the necessary removal of damaged plants may have contributed to the lack of a significant effect. Future studies could avoid this problem by either further increasing the initial sample size, or conducting the experiment under controlled laboratory conditions. It is also possible that the length of time over which this study was conducted was insufficient for detecting differences, and longer-term studies would be valuable in the future. If the experimental results were consistent with those of the carnivorous investment section of this study, we would have expected an interaction between larval presence/absence and the proportion of adult pitchers on a plant. Finally, it is possible that the number of larvae introduced to pitchers at the beginning of the experiment did not remain constant over the course of the study. *Badisis ambulans* does not appear to move among pitchers during the larval stage [27] (personal observations), but future studies could confirm this by continual assessment of the number of larvae present.

It is intriguing that the change in C/N was more variable for plants without larvae (in fact, the s.e. was more than three times higher). While this is not conclusive, it may suggest some form of stabilizing effect of larvae on the change in nutrient status of their host plants. It may be the case, for example, that prey items falling into pitchers over the course of the experiment differed in their digestibility, but that breakdown of prey items by larvae allowed plants to gain similar nutritional benefit from prey, regardless of their initial digestibility. The net influence of this proposed effect on plant fitness is unclear, but these speculations provide scope for new approaches to the study of carnivorous plants and infauna.

It may also be significant that N was the only mineral nutrient we examined directly in this study. Nitrogen is the most commonly measured mineral nutrient in plant nutrition and trophic studies, partly because of the readily available isotopic methods [44,45], and in this case, because it could be measured simultaneously with C in relatively small samples. Nitrogen was therefore used in this study as an indicator of the effect of larvae on overall nutrient status. On the other hand, because the soils in the Southwest Australian Floristic region are phosphorus (P) limited rather than N limited [49–51], some direct measure of P content in plant tissues would be valuable for future studies, if sufficient experimental material were available.

Another possible reason for the failure to detect a significant effect of larvae on C/N is that the percentage of N (approx. 26%) derived from prey is lower in *C. follicularis* than in many other pitcher plants [47], possibly reducing our ability to detect an effect. Furthermore, while the general consensus concerning terrestrial carnivorous plants is that mineral nutrients are assimilated from prey rather than C [1,7,38–43], a few studies have in fact demonstrated movement of C from prey to plants [52,53]. While this is very rare for terrestrial carnivorous plants, as opposed to aquatic ones [54], if *C. follicularis* did in fact take up both C and mineral nutrients from prey, and *B. ambulans* influenced both, then C/N may not be the ideal metric for detecting an effect of infauna on nutrition. Future studies could confirm the precise resources that *C. follicularis* obtains from prey. Finally, more information on the ecology and life history of *B. ambulans* is required. While this species is overwhelmingly the most common macroscopic infauna of *C. follicularis* (personal observations) [27], information on any possible interactions with other species inhabiting the pitchers would be valuable when investigating the effect of *B. ambulans* on its host. Interestingly, we have ourselves made a preliminary discovery of a possible interaction between *B. ambulans* and an as yet unknown species, with consequences for the survival of *C. follicularis* pitchers (see below).

### 4.3. Survival after damage

An unexpected result of this study was that pitchers that had been damaged were significantly less likely to wither if they contained *B. ambulans* larvae. While the sample size for this result was relatively small, it is equivalent to the results of a manipulative experiment and arguably provides stronger evidence of a causal link than our correlative data. One possible explanation for this effect is that after pitchers are damaged and the fluid drains away, the ability of *C. follicularis* to digest prey by itself is severely limited, and it relies almost entirely on breakdown by any larvae that are able to temporarily survive the loss of fluid. Pitchers without larvae are therefore unable to supply the plant with nutrients and are abandoned, whereas pitchers with larvae are maintained for longer. While this explanation makes ecological sense, the system clearly warrants further investigation. In particular, experimental approaches in which deliberately damaged and undamaged plants were assigned to ‘fly’ and ‘no-fly’ treatments would allow for larger sample sizes and greater certainty regarding the cause of pitcher withering.

## 5. Conclusion

The results of this study indicate that the association of infauna with the condition of their host plants can vary depending on the initial context of the host. Future studies into such systems should identify which environmental variables are important for determining the net effect of infauna, and identify possible threshold levels at which the effects of infauna switch from parasitic to mutualistic. The relationship between infauna and relative investment in carnivorous and non-carnivorous structures also warrants further investigation. In particular, experimental approaches would strengthen conclusions regarding causation. The ability of larvae to mediate the effect of additional external stressors (such as pitcher damage) opens exciting new avenues for research into these systems. With regards to this particular system, the apparent context dependence of the relationship of *B. ambulans* to its host means that a system level approach to conservation of this vulnerable plant species may be required [26].

## Supplementary Material

Supplementary Tables 1-6 on additional statistical analyses. Table S1: Proportion of pitcher buds (response) with plant age as a predictor instead of proportion of adult pitchers. Table S2: C/N with plant age. Table S3: Total N with proportion adult pitchers. Table S4: Total N with plant age. Table S5: 15N with proportion adult pitchers. Table S6: 15N with plant age.

## Supplementary Material

R Code provided as supplementary material.

## Data accessibility

All raw data used in this paper are available in the Dryad repository: http://dx.doi.org/10.5061/dryad.p6440 [55]. R code is available as the electronic supplementary material.
