# Supplementary material for: Mutualists or parasites? Context-dependent influence of symbiotic fly larvae on carnivorous investment in the Albany pitcher plant
Source: R Soc Open Sci. 2016 Nov 23;3(11):160690. doi: 10.1098/rsos.160690 (PMC5180157; doi:10.1098/rsos.160690)
Supplement: Supplementary Tables 1-6 on additional statistical analyses. Table S1: Proportion of pitcher buds (response) with plant age as a predictor instead of proportion of adult pitchers. Table S2: C/N with plant age. Table S3: Total N with proportion adult pitchers. Table S4: Total N with plant age. Table  [file rsos160690supp1.docx]

**Supplementary Material**

**Supplementary Table 1** Model comparison of the drivers of variation in the proportion of pitcher buds on *Cephalotus follicularis* plants. Akaike’s Information Criterion (AIC), number of model parameters (*k*) and model weights for binomial generalised linear models within two AIC units of the top model (plus the full model fit for comparison). Predictors were the age (as estimated by the total number of adult carnivorous and non-carnivorous leaves), proportion shading by vegetation (Shading), and density of *Badisis ambulans* larvae (Density). Predictors were standardised prior to model comparisons.

| **Predictors** | ***k*** | **AIC** | **∆AIC** | **Akaike Weight** |
| --- | --- | --- | --- | --- |
| Age + Density + Shading + Age:Density + Density:Shading | 6 | 293.03 | 0.00 | 0.28 |
| Density + Shading + Density:Shading | 4 | 293.19 | 0.16 | 0.26 |
| Age + Shading + Density + Age:Density +Age:Shading + Density:Shading | 7 | 293.98 | 0.95 | 0.18 |
| Age + Density + Shading +Density:Shading | 5 | 294.18 | 1.15 | 0.16 |
| Age + Density + Shading + Age:Density | 5 | 294.79 | 1.76 | 0.12 |
| Full Model: Age × Shading × Density | 8 | 295.72 | 2.69 |  |

**Supplementary Table 2** Results of the best-fit binomial GLM model testing the effects of the proportion of ‘plant age’ (Age), density of the larvae of *Badisis ambulans* (Density) and proportion of shading by neighbouring vegetation (Shading) on the proportion of pitcher buds produced by *Cephalotus follicularis*. See Table 1 for model comparisons. Significant p values are marked *.

| **Predictor** | **Coefficient Estimate** | **SE** | **z** | **p** |
| --- | --- | --- | --- | --- |
| Age | -0.10 | 0.21 | -0.46 | 0.65 |
| Density | -1.15 | 0.33 | -3.52 | 0.0004* |
| Shading | 0.37 | 0.19 | 1.91 | 0.056* |
| Age × Density | 0.95 | 0.57 | 1.64 | 0.10 |
| Density x Shading | 0.89 | 0.43 | 2.06 | 0.040* |

**Supplementary Table 3** Model comparison of the drivers of change in the carbon to nitrogen ratio of *Cephalotus follicularis* tissue from before to eight weeks after experimental manipulation of *Badisis ambulans* larval presence/absence. Akaike’s Information Criterion (AIC), number of model parameters (*k*) and model weight scores for linear models within two AIC units of the top model (plus the full model fit for comparison). Predictors were the total number of adult carnivorous and non-carnivorous leaves (Age – in this analysis this variable replaced the proportion of adult pitchers), proportion shading by vegetation (Shading), pre-experiment density of larvae (Density), and the experimental presence/absence of larvae (Treatment). Predictors were standardised prior to model comparisons.

| **Predictors** | ***k*** | **AIC** | **∆AIC** | **Akaike Weight** |
| --- | --- | --- | --- | --- |
| Null | 1 | 280.41 | 0.00 | 0.40 |
| Shading | 2 | 281.63 | 1.22 | 0.22 |
| Density | 2 | 281.90 | 1.49 | 0.19 |
| Treatment | 2 | 281.96 | 1.56 | 0.19 |
| Full model: Age × Shading × Density × Treatment | 16 | 340.84 | 60.43 |  |

**Supplementary Table 4** Model comparison of the drivers of change in the total nitrogen content of *Cephalotus follicularis* tissue from before to eight weeks after experimental manipulation of *Badisis ambulans* larval presence/absence. Akaike’s Information Criterion (AIC), number of model parameters (*k*) and model weight scores for linear models within two AIC units of the top model (plus the full model fit for comparison). Predictors were the proportion of adult pitchers (PP), proportion shading by vegetation (Shading), pre-experiment density of larvae (Density), and the experimental presence/absence of larvae (Treatment). Predictors were standardised prior to model comparisons.

| **Predictors** | ***k*** | **AIC** | **∆AIC** | **Akaike Weight** |
| --- | --- | --- | --- | --- |
| PP | 2 | -46.28 | 0.00 | 0.30 |
| Null | 1 | -45.54 | 0.74 | 0.20 |
| Density + PP | 3 | -45.34 | 0.94 | 0.18 |
| Shading + PP | 3 | -45.22 | 1.05 | 0.17 |
| Shading | 2 | -44.81 | 1.46 | 0.14 |
| Full model: PP × Shading × Density × Treatment | 16 | 18.47 | 64.75 |  |

**Supplementary Table 5** Model comparison of the drivers of change in the total nitrogen content of *Cephalotus follicularis* tissue from before to eight weeks after experimental manipulation of *Badisis ambulans* larval presence/absence. Akaike’s Information Criterion (AIC), number of model parameters (*k*) and model weight scores for linear models within two AIC units of the top model (plus the full model fit for comparison). Predictors were the total number of adult carnivorous and non-carnivorous leaves (Age – in this analysis this variable replaced the proportion of adult pitchers), proportion shading by vegetation (Shading), pre-experiment density of larvae (Density), and the experimental presence/absence of larvae (Treatment). Predictors were standardised prior to model comparisons.

| **Predictors** | ***k*** | **AIC** | **∆AIC** | **Akaike Weight** |
| --- | --- | --- | --- | --- |
| Null | 1 | -45.54 | 0.00 | 0.47 |
| Shading | 2 | -44.81 | 0.72 | 0.33 |
| Density | 2 | -43.77 | 1.77 | 0.20 |
| Full model: Age × Shading × Density × Treatment | 16 | -14.88 | 60.42 |  |

**Supplementary Table 6** Mean (± standard error) total percentage nitrogen content of *Cephalotus follicularis* pitchers before and eight months after experimental manipulation of *Badisis ambulans* presence/absence. “Fly” pitchers had all *B. ambulans* larvae removed and then 10 added to a single pitcher, while “no fly” plants had all larvae removed and none added.

|  | **Before** | **After** |
| --- | --- | --- |
| **Fly** | 0.40±0.01 | 0.48±0.02 |
| **No Fly** | 0.41±0.02 | 0.47±0.03 |

**Supplementary Table 7** Model comparison of the drivers of change in δ^15^N of *Cephalotus follicularis* tissue eight weeks after experimental manipulation of *Badisis ambulans* larval presence/absence. A measure of δ^15^N before experimental manipulation was not possible as the required quantity of tissue could not be collected without damaging the plant. Akaike’s Information Criterion (AIC), number of model parameters (*k*) and model weight scores for linear models within two AIC units of the top model (plus the full model fit for comparison). Predictors were the proportion of adult pitchers (PP), proportion shading by vegetation (Shading), pre-experiment density of larvae (Density), and the experimental presence/absence of larvae (Treatment). Predictors were standardised prior to model comparisons.

| **Predictors** | ***k*** | **AIC** | **∆AIC** | **Akaike Weight** |
| --- | --- | --- | --- | --- |
| Density + PP + Density:PP | 4 | 120.34 | 0.00 | 0.36 |
| Null | 1 | 120.44 | 0.09 | 0.34 |
| Treatment + Density + PP +Density:PP | 5 | 121.87 | 1.52 | 0.17 |
| PP | 2 | 122.31 | 1.96 | 0.13 |
| Full model: PP × Shading × Density × Treatment | 16 | 185.03 | 64.69 |  |

**Supplementary Table 8** Model comparison of the drivers of δ^15^N content of *Cephalotus follicularis* tissue eight weeks after experimental manipulation of *Badisis ambulans* larval presence/absence. Akaike’s Information Criterion (AIC), number of model parameters (*k*) and model weight scores for linear models within two AIC units of the top model (plus the full model fit for comparison). Predictors were the total number of adult carnivorous and non-carnivorous leaves (Age – in this analysis this variable replaced the proportion of adult pitchers), proportion shading by vegetation (Shading), pre-experiment density of *Badisis ambulans* larvae (Density), and the experimental presence/absence of larvae (Treatment). Predictors were standardised prior to model comparisons.

| **Predictors** | ***k*** | **AIC** | **∆AIC** | **Akaike Weight** |
| --- | --- | --- | --- | --- |
| Null | 1 | 120.44 | 0.00 | 0.57 |
| Age | 2 | 122.36 | 1.92 | 0.22 |
| Shading | 2 | 122.43 | 1.99 | 0.21 |
| Full model: Age × Shading × Density × Treatment | 16 | 187.65 | 67.21 |  |

**Supplementary Table 9** Mean (± standard error) δ^15^N isotope ratios of *Cephalotus follicularis* pitchers eight months after experimental manipulation of *Badisis ambulans* presence/absence. “Fly” pitchers had all *B. ambulans* larvae removed and then 10 added to a single pitcher, while “no fly” plants had all larvae removed and none added.

|  | **δ^15^N** |
| --- | --- |
| **Fly** | 1.54±0.40 |
| **No Fly** | 1.28±0.48 |
